# Epicardial Fat Thickness as a Marker of Coronary Artery Disease in Diabetics: A Single Center Study

**DOI:** 10.1002/clc.70171

**Published:** 2025-07-05

**Authors:** Abdul Nadeem Akhter, Fnu Aisha, Aimen Binte Moazzam, Sardar Humayun Babar Khan, Jahanzeb Malik, Abida Parveen

**Affiliations:** ^1^ Department of Medicine Ibn e Seena Hospital Kabul Afghanistan

**Keywords:** CAD severity, cardiovascular disease, coronary artery disease, echocardiography, epicardial fat thickness, noninvasive marker, risk stratification, type 2 diabetes mellitus

## Abstract

**Background:**

Epicardial fat thickness (EFT) is a visceral fat depot with pro‐inflammatory properties, located adjacent to coronary vessels, and has been proposed as a marker of coronary artery disease (CAD). This study aimed to evaluate the association between EFT and the presence and severity of CAD in patients with type 2 diabetes mellitus.

**Methods:**

This retrospective study was conducted at the Abbas Institute of Medical Sciences (AIMS) between January 2020 and March 2025 (Study ID: AIMS/25/007). A total of 2340 diabetic patients (mean age: 58.3 ± 9.6 years) were included. EFT was measured using transthoracic echocardiography, and CAD presence and severity were assessed via coronary angiography. Logistic regression analysis was used to evaluate associations, with results expressed as odds ratios (OR) with 95% confidence intervals (CI).

**Results:**

Elevated EFT (≥ 5 mm) was observed in 1281 patients (54.7%). CAD was present in 1121 individuals (47.9%), with significantly higher rates in those with elevated EFT (65.7% vs. 26.3%, *p* < 0.001). EFT ≥ 5 mm was associated with a 5.38‐fold increased odds of CAD (95% CI: 4.59–6.30, *p* < 0.001). Moreover, patients with elevated EFT had a significantly higher prevalence of multi‐vessel CAD, indicating a correlation between EFT and disease severity.

**Conclusions:**

In diabetic patients, elevated EFT is significantly associated with both the presence and severity of CAD. EFT measurement via echocardiography may serve as a simple, noninvasive tool for cardiovascular risk stratification and early intervention planning.

## 1 Introduction

Cardiovascular disease remains the leading cause of morbidity and mortality among individuals with diabetes mellitus (DM), with coronary artery disease (CAD) representing the most prevalent and serious manifestation [1]. The interplay between chronic hyperglycemia, insulin resistance, and systemic inflammation contributes significantly to the accelerated development of atherosclerosis in diabetic patients. Early detection and accurate risk stratification of CAD in this population are crucial to mitigate adverse outcomes [2]. However, traditional risk assessment tools often fall short in capturing the unique pathophysiological mechanisms at play in diabetic individuals, underscoring the need for novel, reliable, and noninvasive biomarkers [3].

Epicardial adipose tissue (EAT), a form of visceral fat located between the myocardium and the visceral pericardium, has emerged as a potential marker of cardiovascular risk. Unlike other fat depots, EAT is metabolically active and exerts paracrine and vasocrine effects on adjacent coronary arteries due to the absence of a fascial barrier [4]. It secretes a range of pro‐inflammatory cytokines and adipokines that can promote endothelial dysfunction, plaque formation, and vascular inflammation—key processes in the pathogenesis of CAD [5]. Studies suggest that epicardial fat thickness (EFT), measurable via echocardiography, computed tomography, or magnetic resonance imaging, correlates with the presence and severity of CAD, particularly in individuals with diabetes [6].

Despite growing interest in the clinical utility of EFT, limited original research has focused specifically on its role as a diagnostic or prognostic marker in diabetic populations [7]. This study aims to evaluate the relationship between epicardial fat thickness and coronary artery disease in patients with type 2 diabetes mellitus. By assessing EFT in correlation with angiographic evidence of CAD, this study seeks to establish EFT as a practical, noninvasive indicator that may enhance cardiovascular risk assessment and inform targeted prevention strategies in diabetic care.

## 2 Methods

### 2.1 Study Design and Setting

This was a retrospective study conducted at the Department of Cardiology, Abbas Institute of Medical Sciences (AIMS), over a 5‐year period from January 2020 to March 2025. The study was approved by the institutional ethical review board under the study identification number AIMS/25/007. All participants provided written informed consent before enrollment in accordance with the Declaration of Helsinki.

### 2.2 Study Population

A total of 2340 patients with type 2 diabetes mellitus were recruited consecutively from the outpatient and inpatient departments of AIMS. Inclusion criteria consisted of adults aged 30–75 years with a confirmed diagnosis of type 2 diabetes mellitus for at least 1 year. Patients with known coronary artery disease (history of myocardial infarction, previous coronary intervention, or coronary bypass surgery), structural heart disease, pericardial disease, or systemic inflammatory conditions were excluded. Pregnant women and individuals with poor echocardiographic windows were also excluded from the study.

### 2.3 Data Collection and Clinical Assessment

Detailed demographic and clinical data were obtained through structured interviews and review of medical records. Information collected included age, sex, duration of diabetes, smoking status, body mass index (BMI), blood pressure, and lipid profile. All participants underwent a comprehensive cardiovascular examination, and baseline laboratory investigations were recorded. The presence of hypertension and dyslipidemia was defined according to standard international guidelines.

### 2.4 Measurement of Epicardial fat Thickness

**FIGURE 1:**
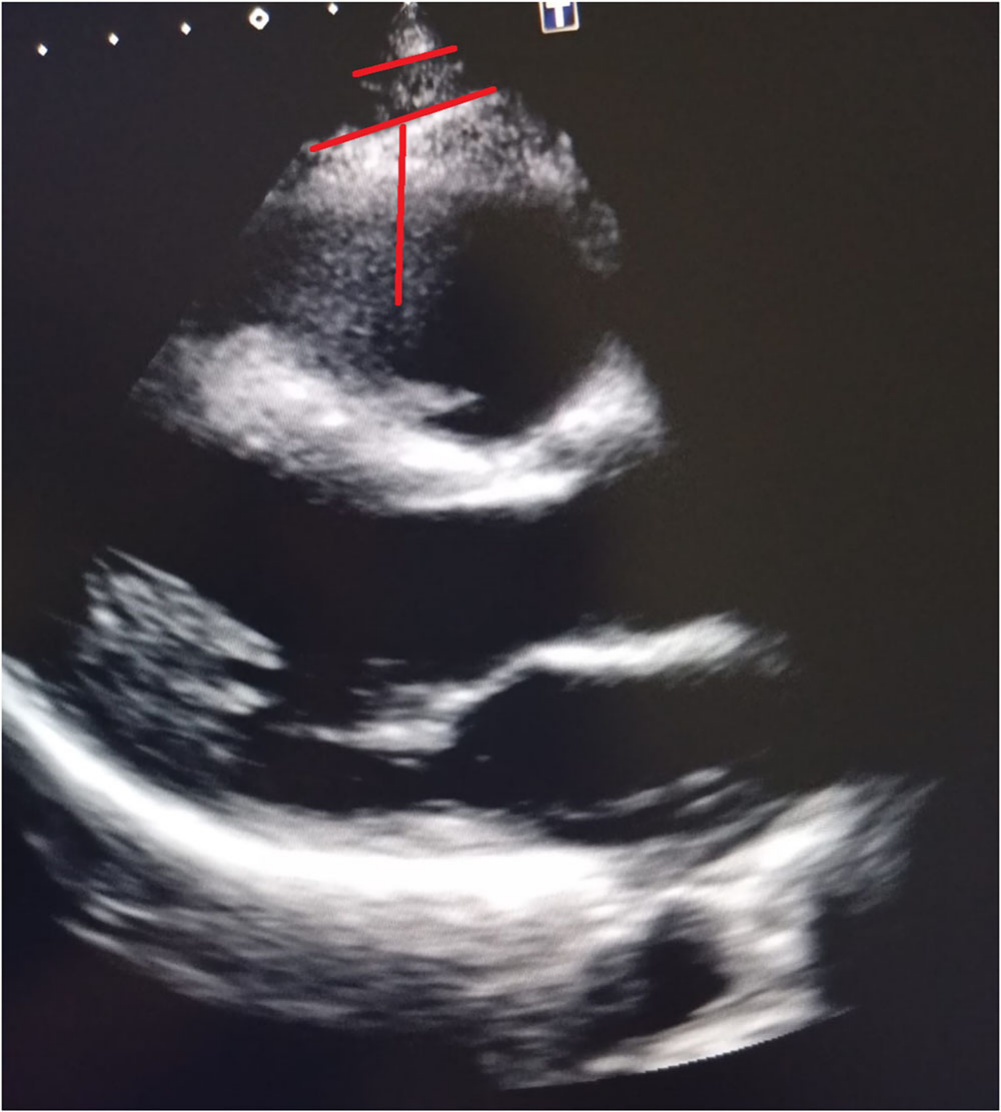
Measurements of epicardial fat thickness through transthoracic echocardiography. In the long‐axis parasternal view of a transthoracic echocardiogram, the red lines indicates epicardial fat located over the free wall of the right ventricle. It appears as an echo‐free space between the outer myocardial wall and the visceral layer of the pericardium.

Epicardial fat thickness (EFT) was measured using transthoracic echocardiography (TTE) with a commercially available system and a 2.5–3.5 MHz transducer. All measurements were performed by a single experienced cardiologist to minimize interobserver variability. EFT was defined as the echo‐free space between the outer wall of the myocardium and the visceral layer of the pericardium. Measurements were taken in the parasternal long‐axis view at end‐systole, perpendicular to the right ventricular free wall as shown in Figure 1. The average of three cardiac cycles was used for analysis. An EFT value ≥ 5 mm was considered elevated based on previous literature [8].

### 2.5 Assessment of Coronary Artery Disease

All patients underwent either noninvasive coronary imaging or invasive coronary angiography, based on clinical indications. CAD was defined as the presence of ≥ 50% luminal diameter stenosis in at least one major epicardial coronary artery. The severity of CAD was further categorized as single‐vessel, double‐vessel, or triple‐vessel disease. The imaging studies were interpreted by interventional cardiologists who were blinded to the EFT measurements.

### 2.6 Statistical Analysis

Data were analyzed using SPSS version 25.0 (IBM Corp., Armonk, NY, USA). Continuous variables were presented as mean ± standard deviation, while categorical variables were expressed as frequencies and percentages. The association between EFT and the presence of CAD was assessed using chi‐square tests for categorical variables and independent t‐tests for continuous variables. Logistic regression analysis was performed to determine the predictive value of EFT for CAD after adjusting for potential confounders. A *p*‐value < 0.05 was considered statistically significant.

## 3 Results

A total of 2340 patients with type 2 diabetes mellitus were enrolled in this study. The mean age of the study population was 58.3 ± 9.6 years, with males comprising 60.7% (*n* = 1421) of participants. Elevated epicardial fat thickness (EFT ≥ 5 mm) was observed in 1281 individuals (54.7%), while coronary artery disease (CAD) was diagnosed in 1121 patients (47.9%) based on angiographic and noninvasive imaging findings (Table 1).

**TABLE 1:** Baseline characteristics of the study population (*N* = 2340).

Variable	Mean ± SD/n (%)
Age (years)	58.3 ± 9.6
Sex	
− Male	1421 (60.7%)
− Female	919 (39.3%)
Body Mass Index (kg/m²)	27.5 ± 3.8
Duration of Diabetes (years)	8.2 ± 4.5
Hypertension	1651 (70.5%)
Dyslipidemia	1480 (63.2%)
Smoking Status	
− Current/Former Smokers	761 (32.5%)
− Non‐smokers	1579 (67.5%)
Systolic BP (mmHg)	136.2 ± 14.7
Diastolic BP (mmHg)	84.1 ± 9.3
Fasting Blood Glucose (mg/dL)	146.7 ± 38.2
HbA1c (%)	7.9 ± 1.3
Total Cholesterol (mg/dL)	198.4 ± 35.9
LDL‐C (mg/dL)	122.6 ± 29.7
HDL‐C (mg/dL)	43.8 ± 9.1
Triglycerides (mg/dL)	174.5 ± 52.3
Elevated EFT (≥ 5 mm)	1281 (54.7%)
Presence of CAD	1121 (47.9%)

The presence of CAD was significantly higher among patients with elevated EFT (65.7%) compared to those with EFT < 5 mm (26.3%), demonstrating a strong association between EFT and CAD (*p* < 0.001). Unadjusted logistic regression analysis revealed that patients with elevated EFT had more than five times higher odds of having CAD compared to those with normal EFT (OR 5.38; 95% CI: 4.59–6.30; *p* < 0.001). Other significant predictors of CAD included male sex (OR 1.47; 95% CI: 1.25–1.73), hypertension (OR 2.48; 95% CI: 2.08–2.95), dyslipidemia (OR 2.35; 95% CI: 2.00–2.76), and current or former smoking (OR 1.78; 95% CI: 1.49–2.14) (Table 2).

**TABLE 2:** Association between epicardial fat thickness and coronary artery disease.

Variable	CAD Present (*n* = 1121)	CAD Absent (*n* = 1219)	Odds Ratio (OR)	95% CI	*p*‐value
EFT ≥ 5 mm	842 (75.1%)	439 (36.0%)	5.38	4.59–6.30	< 0.001
Male Sex	735 (65.5%)	686 (56.3%)	1.47	1.25–1.73	< 0.001
Hypertension	896 (79.9%)	755 (61.9%)	2.48	2.08–2.95	< 0.001
Dyslipidemia	823 (73.4%)	657 (53.9%)	2.35	2.00–2.76	< 0.001
Smoking (Current/Former)	438 (39.1%)	323 (26.5%)	1.78	1.49–2.14	< 0.001
BMI ≥ 30 kg/m²	321 (28.6%)	278 (22.8%)	1.36	1.12–1.65	0.001
HbA1c ≥ 8%	544 (48.5%)	419 (34.4%)	1.78	1.51–2.11	< 0.001

When participants were stratified by EFT status, those with elevated EFT (≥ 5 mm) were found to have significantly worse clinical and metabolic profiles. These patients were older (60.2 ± 9.3 vs. 55.8 ± 8.9 years), had higher BMI (29.1 ± 4.1 vs. 25.4 ± 3.2 kg/m²), and showed significantly elevated levels of HbA1c (8.3 ± 1.4 vs. 7.4 ± 1.1%), systolic blood pressure (140.1 ± 14.2 vs. 131.2 ± 13.5 mmHg), LDL cholesterol (128.9 ± 30.3 vs. 115.6 ± 27.1 mg/dL), and triglycerides (186.2 ± 55.6 vs. 159.3 ± 48.2 mg/dL) compared to those with EFT < 5 mm (all *p* < 0.001) (Table 3).

**TABLE 3:** Comparison of clinical and biochemical parameters based on EFT status.

Parameter	EFT < 5 mm (*n* = 1059)	EFT ≥ 5 mm (*n* = 1281)	*p*‐value
Age (years)	55.8 ± 8.9	60.2 ± 9.3	< 0.001
Male (%)	592 (55.9%)	829 (64.7%)	< 0.001
BMI (kg/m²)	25.4 ± 3.2	29.1 ± 4.1	< 0.001
HbA1c (%)	7.4 ± 1.1	8.3 ± 1.4	< 0.001
Systolic BP (mmHg)	131.2 ± 13.5	140.1 ± 14.2	< 0.001
LDL‐C (mg/dL)	115.6 ± 27.1	128.9 ± 30.3	< 0.001
Triglycerides (mg/dL)	159.3 ± 48.2	186.2 ± 55.6	< 0.001
Presence of CAD (%)	279 (26.3%)	842 (65.7%)	< 0.001

The Table 4 presents the distribution of coronary artery disease (CAD) severity and specific vessel involvement in patients stratified by epicardial fat thickness (EFT). Among patients with EFT < 5 mm (*n* = 1059), the majority (73.6%) had no evidence of CAD, compared to only 34.3% of those with EFT ≥ 5 mm (*n* = 1281), indicating a significant inverse relationship between EFT and absence of CAD (*p* < 0.001). Conversely, the prevalence of single‐, double‐, and triple‐vessel disease increased markedly with higher EFT.

**TABLE 4:** Severity of CAD by EFT category.

CAD Severity	EFT < 5 mm (*n* = 1059)	EFT ≥ 5 mm (*n* = 1281)	*p*‐value
No CAD	780 (73.6%)	439 (34.3%)	< 0.001
Single‐vessel disease	179 (16.9%)	416 (32.5%)	< 0.001
− LAD	93 (8.8%)	226 (17.6%)	< 0.001
− OM	41 (3.9%)	98 (7.7%)	< 0.001
− PDA	30 (2.8%)	71 (5.5%)	< 0.01
− LM	15 (1.4%)	21 (1.6%)	0.44
Double‐vessel disease	65 (6.1%)	256 (20.0%)	< 0.001
− LAD + OM	29 (2.7%)	96 (7.5%)	< 0.001
− LAD + PDA	21 (2.0%)	88 (6.9%)	< 0.001
− OM + PDA	15 (1.4%)	72 (5.6%)	< 0.001
Triple‐vessel disease	35 (3.3%)	170 (13.3%)	< 0.001
− LAD + OM + PDA	28 (2.6%)	140 (10.9%)	< 0.001
− LAD + OM + LM	7 (0.7%)	30 (2.3%)	< 0.01

In the EFT ≥ 5 mm group, 32.5% of patients had single‐vessel disease, significantly higher than the 16.9% observed in those with EFT < 5 mm (*p* < 0.001). Within the single‐vessel category, involvement of the left anterior descending artery (LAD) was most common (17.6% vs. 8.8%), followed by the obtuse marginal (OM) artery (7.7% vs. 3.9%) and posterior descending artery (PDA) (5.5% vs. 2.8%). Left main (LM) artery involvement as a single‐vessel lesion was rare and not significantly different between groups (1.6% vs. 1.4%).

Double‐vessel disease was found in 20.0% of patients with higher EFT, significantly more than the 6.1% in the lower EFT group (*p* < 0.001). The most frequent combinations were LAD + OM (7.5% vs. 2.7%), LAD + PDA (6.9% vs. 2.0%), and OM + PDA (5.6% vs. 1.4%), all showing significant associations with increased EFT.

Triple‐vessel disease was present in 13.3% of patients with EFT ≥ 5 mm, compared to only 3.3% in those with EFT < 5 mm (*p* < 0.001). The most common pattern was combined involvement of LAD, OM, and PDA (10.9% vs. 2.6%), with some cases also involving the LM artery (2.3% vs. 0.7%).

Overall, these findings demonstrate that increased epicardial fat thickness is significantly associated with greater severity of CAD and with more extensive involvement of major coronary arteries, particularly the LAD, OM, and PDA. The results support the role of EFT as a potential noninvasive marker for predicting CAD burden and distribution.

## 4 Discussion

This study evaluated the role of epicardial fat thickness (EFT) as a potential marker for coronary artery disease (CAD) in a large cohort of patients with type 2 diabetes mellitus. Our findings demonstrate a strong and statistically significant association between elevated EFT (≥ 5 mm) and both the presence and severity of CAD. Patients with increased EFT were more likely to be older, male, hypertensive, dyslipidemic, and have poorer glycemic control, highlighting a clustering of traditional cardiovascular risk factors alongside elevated EFT. Importantly, EFT emerged as an independent and powerful predictor of CAD in this diabetic population, with an unadjusted odds ratio of 5.38 (95% CI: 4.59–6.30, *p* < 0.001).

Epicardial fat is a metabolically active visceral fat depot that lies in close proximity to coronary arteries and shares the same microcirculation. It secretes various pro‐inflammatory and pro‐atherogenic adipokines, such as interleukin‐6, tumor necrosis factor‐alpha, and resistin, which can promote endothelial dysfunction and atherosclerosis [9]. Our findings are consistent with earlier studies that have identified increased EFT as a marker of subclinical atherosclerosis and an independent predictor of CAD, particularly in high‐risk groups such as patients with metabolic syndrome and diabetes. However, our study adds to the existing body of evidence by including a larger diabetic population and by establishing a clear relationship between EFT and CAD severity.

Moreover, the progressive increase in the prevalence of single, double, and triple‐vessel disease with increasing EFT suggests that EFT may not only reflect the presence of CAD but also the extent of coronary involvement. These findings support the utility of EFT measurement as a noninvasive, accessible, and cost‐effective tool in cardiovascular risk stratification. Measurement of EFT via echocardiography, as used in our study, can be easily incorporated into routine cardiac evaluation, especially in diabetic patients who often require regular cardiovascular screening.

Despite the strength of our findings, several limitations should be acknowledged. First, the cross‐sectional nature of the study limits the ability to establish causality between increased EFT and CAD. Second, while EFT was measured using echocardiography, more precise imaging modalities such as cardiac CT or MRI could provide more accurate volumetric assessments. Third, although we adjusted for several conventional risk factors, the influence of unmeasured confounders cannot be excluded.

### 4.1 Future Directions

Given the strong association between epicardial fat thickness (EFT) and coronary artery disease (CAD) observed in this study, future research should focus on prospective, longitudinal studies to determine whether elevated EFT can predict future cardiovascular events in diabetic populations [10]. Additionally, interventional studies exploring whether reductions in EFT—through lifestyle modifications, pharmacotherapy, or bariatric interventions—can lead to a decreased incidence or progression of CAD would provide valuable clinical insights [11]. Further investigations using advanced imaging modalities such as cardiac MRI or CT could also enhance the precision of EFT measurement and validate echocardiographic cut‐off values. Integrating EFT assessment into existing cardiovascular risk models may refine risk stratification and help guide personalized treatment strategies in diabetic patients [12].

Our study offers high incremental value over existing literature by providing a large‐scale, well‐characterized analysis of the relationship between epicardial fat thickness (EFT) and both the presence and angiographic severity of coronary artery disease (CAD) specifically in patients with type 2 diabetes mellitus—a high‐risk yet underexplored population in this context. Unlike prior studies that often focused on either EFT or CAD in isolation, our study comprehensively integrates detailed echocardiographic measurement of EFT with coronary imaging findings, including vessel‐specific analysis of single‐, double‐, and triple‐vessel disease [13]. This stratification not only strengthens the clinical relevance of EFT as a marker of atherosclerotic burden but also identifies specific coronary territories most affected by increased EFT. Additionally, the robust sample size, uniform measurement methodology, and multivariable regression analysis enhance the reliability and generalizability of your findings. Collectively, this study supports the utility of EFT as a noninvasive, easily accessible marker for CAD risk stratification in diabetic patients, potentially informing earlier detection and more targeted prevention strategies.

## 5 Conclusion

This study demonstrates that elevated epicardial fat thickness is significantly associated with the presence and severity of coronary artery disease in patients with type 2 diabetes mellitus. EFT, as a noninvasive and easily obtainable echocardiographic marker, may serve as a valuable tool for early detection and risk stratification of CAD in this high‐risk population. Incorporating EFT assessment into routine clinical practice may enhance cardiovascular screening and inform timely preventive and therapeutic interventions.

## Data Availability

Data sharing is not applicable to this article as no new data were created or analyzed in this study.

## References

[1] D. Aronson and E. R. Edelman , “Coronary Artery Disease and Diabetes Mellitus,” Cardiology Clinics 32, no. 3 (2014): 439–455, 10.1016/j.ccl.2014.04.001. 25091969 PMC4672945

[2] T. Li , P. Wang , X. Wang , et al., “Inflammation and Insulin Resistance in Diabetic Chronic Coronary Syndrome Patients,” Nutrients 15, no. 12 (2023): 2808, 10.3390/nu15122808. 37375712 PMC10301506

[3] B. Buijsse , R. K. Simmons , S. J. Griffin , and M. B. Schulze , “Risk Assessment Tools for Identifying Individuals at Risk of Developing Type 2 Diabetes,” Epidemiologic Reviews 33, no. 1 (2011): 46–62, 10.1093/epirev/mxq019. 21622851 PMC3132807

[4] G. Iacobellis , “Epicardial Adipose Tissue in Contemporary Cardiology,” Nature Reviews Cardiology 19, no. 9 (2022): 593–606, 10.1038/s41569-022-00679-9. 35296869 PMC8926097

[5] H. Zhang and N. S. Dhalla , “The Role of Pro‐Inflammatory Cytokines in the Pathogenesis of Cardiovascular Disease,” International Journal of Molecular Sciences 25, no. 2 (2024): 1082, 10.3390/ijms25021082. 38256155 PMC10817020

[6] S. K. Shambu , N. Desai , N. Sundaresh , M. S. Babu , B. Madhu , and O. J. Gona , “Study of Correlation Between Epicardial Fat Thickness and Severity of Coronary Artery Disease,” Indian Heart Journal 72, no. 5 (2020): 445–447, 10.1016/j.ihj.2020.07.014. 33189210 PMC7670255

[7] M. Ortiz‐Martínez , M. González‐González , A. J. Martagón , V. Hlavinka , R. C. Willson , and M. Rito‐Palomares , “Recent Developments in Biomarkers for Diagnosis and Screening of Type 2 Diabetes Mellitus,” Current diabetes reports 22, no. 3 (2022): 95–115, 10.1007/s11892-022-01453-4. 35267140 PMC8907395

[8] F. Mookadam , R. Goel , M. Alharthi , P. Jiamsripong , and S. Cha , “Epicardial fat and Its Association With Cardiovascular Risk: A Cross‐Sectional Observational Study,” Heart Views : The Official Journal of the Gulf Heart Association 11, no. 3 (2010): 103–108, 10.4103/1995-705X.76801. 21577377 PMC3089830

[9] G. Iacobellis and A. C. Bianco , “Epicardial Adipose Tissue: Emerging Physiological, Pathophysiological and Clinical Features,” Trends in Endocrinology & Metabolism 22, no. 11 (2011): 450–457, 10.1016/j.tem.2011.07.003. 21852149 PMC4978122

[10] A. Rostamzadeh , K. Khademvatani , M. H. Seyed Mohammadzadeh , et al., “Association of Epicardial fat Thickness Assessed by Echocardiography With the Severity of Coronary Artery Disease,” Journal of Cardiovascular and Thoracic Research 12, no. 2 (2020): 114–119, 10.34172/jcvtr.2020.19. 32626551 PMC7321005

[11] P. Gaudel , S. Neupane , A. M. Koivisto , M. Kaunonen , and A. Rantanen , “Effects of Intervention on Lifestyle Changes Among Coronary Artery Disease Patients: A 6‐Month Follow‐up Study,” Nursing Open 9, no. 4 (2022): 2024–2036, 10.1002/nop2.1212. 35434911 PMC9190674

[12] F. Perone , M. Bernardi , A. Redheuil , et al., “Role of Cardiovascular Imaging in Risk Assessment: Recent Advances, Gaps in Evidence, and Future Directions,” Journal of Clinical Medicine 12, no. 17 (2023): 5563, 10.3390/jcm12175563. 37685628 PMC10487991

[13] K. Meenakshi , M. Rajendran , S. Srikumar , and S. Chidambaram , “Epicardial Fat Thickness: A Surrogate Marker of Coronary Artery Disease ‐ Assessment by Echocardiography,” Indian Heart Journal 68, no. 3 (2016): 336–341, 10.1016/j.ihj.2015.08.005. 27316487 PMC4911429

